# Molecular Characterization of a Fungus Producing Membrane Active Metabolite and Analysis of the Produced Secondary Metabolite

**DOI:** 10.29252/.23.2.121

**Published:** 2019-03

**Authors:** Parisa Azerang, Vahid Khalaj, Farzad Kobarfard, Parviz Owlia, Soroush Sardari, Sahar Shahidi

**Affiliations:** 1 Drug Design and Bioinformatics Unit, Medical Biotechnology Department, Biotechnology Research Center, Pasteur Institute of Iran, Tehran, Iran;; 2 Medical Biotechnology Departments, Biotechnology Research Center, Pasteur Institute of Iran, Tehran, Iran;; 3 Department of Medicinal Chemistry, School of Pharmacy, Shahid Beheshti University of Medical Sciences, Tehran, Iran;; 4 Molecular Microbiology Research Center, Faculty of Medicine, Shahed University, Tehran, Iran

**Keywords:** *Aspergillus*, Membrane activity, Natural products

## Abstract

**Background:**

The majority of studies on soil *Aspergillus* concern the isolation and characterization of the antimicrobial compounds produced by this organism. Our previous studies indicated an isolated *Aspergillus* strain soil to be of interest, and this subject is further investigated here.

**Method:**

Soil samples of various locations in Iran were collected. Extract from *Aspergillus *sp. culture was obtained using ethyl acetate fractionation. Antimicrobial activity testing was performed using broth microdilution assay against *Escherichia coli*, *Candida albicans*, and *Staphylococcus aureus* microorganisms. One metabolite PA3-d10 was isolated from these active extracts and identified using thin layer chromatography, preparative thin-layer chromatography, HPLC, ^1^HNMR (proton nuclear magnetic resonance), 2D NMR, and LC-MS (liquid chromatography-mass spectrometry).

**Results:**

According to morphological and biochemical properties as well as ITS rDNA sequencing, we identified an isolate of *Aspergillus flavus*. The ethyl acetate fraction of the fermentation medium containing membrane active metabolites showed antimicrobial effects against different bacterial and yeast indicator strains. One metabolite from these active extracts was finally identified.

**Conclusion:**

Membrane active fraction produced by *Aspergillus* strain in this research demonstrated antimicrobial activities against bacteria and yeast strains. Therefore, this metabolite can be considered as a potential antimicrobial membrane active agent.

## INTRODUCTION

Drug-resistant pathogens like bacteria, parasitic protozoans, and fungi are responsible for a number of diseases^[^^1^^]^. The emergence of multiple drug-resistant microbes in recent times creates the need to discover novel antimicrobials for the treatment of human diseases^[^^2^^]^. Many research groups are currently involved in the search for newer and more effective agents to deal with these pathogens. More than 60% of all the drugs are obtained from natural products and their derivatives^[^^3^^]^. Microorganisms have the ability to produce antimicrobial compounds as secondary metabolites, which are important for survival in their complex ecosystems.

Only 1% of bacteria and 5% of fungi have been characterized so far, and the majority of these microbes remain unidentified, especially with regard to their roles in human disease^[^^4^^]^. In addition, more than 50% of the anticancer drugs and nearly 70% of the antimicrobial medicines currently used in clinic are derived from natural products^[^^5^^]^. A number of studies have screened antibiotics producing soil fungi to discover novel pharmaceutical substrates^[^^6^^,^^7^^]^.

Antibiotics have various modes of action like inhibition of protein synthesis, cell wall formation, as well as nucleic and ribonucleic acid synthesis^[^^8^^,^^9^^]^. The bacterial cell membrane is a potential antibiotic target and presents a lower probability of resistance development. Antibiotics that anchor themselves in the cell membrane seem to be more potent due to their fast and extensive antimicrobial effects and are possibly less prone to be selected for resistance^[^^10^^]^. In our previous study, we isolated 60 species of fungi from the soil samples that were collected from different areas in Iran, including desert, forest, farming land, and mineral soils^[^^11^^]^. Twenty isolates were effective against indicator strains, including *Candida albicans* ATCC 10231, *S. aureus* ATCC 25923, and *Escherichia coli* ATCC 25922. Only one fungus targeted the membrane of these indicator strains. Fungal colonies were identified according to morphological characteristics, and the isolate was assigned to the genus *Aspergillus*. In this report, we identified the membrane-active metabolites produced by this *Aspergillus *strain using bioassays and evaluated them with a vesicle membrane model^[^^12^^,^^13^^]^.

## MATERIALS AND METHODS

**Morphological studies by slide culture preparation**

***Culture and identification***

*Aspergillus* isolate was identified at the genus level by colony morphology on malt extract agar. To identify the isolate at the species level, the Czapek Dox agar medium was used. The colonies were identified on the basis of major macroscopic features like colony diameter, color of conidia, as well as reverse, exudates, and colony texture. The isolates were studied microscopically using the classic slide culture method. A 20 × 20 mm coverslip was pressed into the agar medium and carefully removed when the molds peculated and mounted in a drop of lacto-fuchsine on a microscope slide. Microscopic identification features included stipes, color, length and shape of vesicles, conidial heads, and metula covering and shape. The morphological characteristics of the isolates were compared with those of the established species^[^^14^^]^.

**Identification and characterization of** ***Aspergillus*** ***DNA extraction***

DNA from *Aspergillus* was extracted as described below. Briefly, the fungus was inoculated in a 5-mL Sabouraud broth (2% glucose w/v and 1% peptone w/v) supplemented with chloramphenicol and incubated in a shaker at 120 rpm at 30 °C overnight. After centrifugation, the mycelium was recovered and washed with 0.9% (w/v) NaCl, frozen in liquid nitrogen and ground to a fine powder. The powder was incubated in 500 µL extraction buffer (50 mM of EDTA, 50 mM of Tris-HCl, 1% 2-mercaptoethanol, and 3% SDS) at 65 °C for 20 min. The lysate was extracted once with an equal volume of phenol/ chloroform (1:1, v/v) and once with chloroform/ isoamyl alcohol (24:1 v/v). The genomic DNA was then precipitated by isopropanol. The DNA pellet was washed with 70% (v/v) ethanol, dried in vacuum and re-suspended in TE buffer (10 mM of Tris-HCl, 1 mM of EDTA, pH 8). The DNA was further cleaned up with GeneAll Expin Combo (Geneall Biotechnology Co. Ltd, South Korea) according to the manufacturer's instructions^[^^15^^,^^16^^]^.

***PCR amplification***

ITS1-5.8S and ITS2 rDNA were PCR-amplified using a Perkin Elmer 2400 thermal cycler (Perkin Elmer Cetus Corporation, Emeryville, CA, USA). The primer pairs ITS5 (3´GGAAGTAAAAGTCGTAACA AGG5´) and ITS4 (3´TCCTCCGCTTATTGATA TGC 5´) have been described in a previous study^[17]^. The reaction mix consisted of 12.5 µL of 2X ready-to-use master mix (Sinaclone, Iran), 1 µL of each primer (10 pmol; Sinaclone, Iran), 2 µL of DNA, and 6.5 µL of distilled water to a total volume of 25 µL. The PCR condition was as follows: pre-denaturation at 95 °C for 5 min, 35 cycles of denaturation at 95 °C for 30 s, annealing at 50 °C for 1 min, and extension at 72 °C for 40 s, and a final extension of 5 min at 72 ^o^C. The amplified fragments were analyzed by 1% agarose gel electrophoresis in 1 TEB buffer against 1-kb DNA ladder (Fermentas, USA), and the bands visualized under a UV light.

***Sequence analysis***

The amplified region was sequenced and compared with the sequences available in the data bank of the National Center for Biotechnology and Information (NCBI), using BLAST search tool^[^^18^^]^. The species was identified based on the best score.

**Growth conditions** **and ethyl acetate extraction**

The isolated strain of *Aspergillus* was grown in a static condition in malt extract agar medium at 37 °C for 14 days. After the incubation period, a loop of spores was scraped from the plate and inoculated into 20 mL malt extract broth. The flask was placed in a shaker incubator and rotated at 150 rpm at 27 °C for seven days. A 10-mL inoculum from this initial culture was seeded to 1000 mL Erlenmeyer flasks containing 500 mL of malt extract broth and incubated at 200 rpm at 37 °C for 14 days. The culture broth was centrifuged at 2000 ×g for 30 min, and the biomass was discarded. The broth was filtered to remove any remaining cells, and ethyl acetate was added to the aqueous phase at a ratio of 1:1 (v/v) three times. The mixture was shaken to extract the lipid soluble membrane active fraction into the ethyl acetate phase and then evaporated. The residue obtained was tested for antimicrobial activity^[^^19^^]^.

**Determination of minimum inhibitory concentration (MIC)**

MIC of the ethyl acetate extract was tested against one species of Gram-positive and of Gram-negative bacteria and one fungal species. *E. coli *ATCC 25922, *C. albicans* ATCC 10231, and *Staphylococcus aureus *ATCC 25923 were used as the test strains. The ethyl acetate fraction of *Aspergillus* was dissolved in DMSO at the concentration of 10 mg/mL. The broth microdilution method was applied for testing antifungal and antimicrobial activities based on NCCLS M27-A^[^^20^^]^. Sabouraud maltose broth was used as the growth medium for *C. albicans*, and *E. coli *and* S. aureus *were cultured in Muller Hilton Broth. Amphotericin B was used as a reference in the antifungal test and streptomycin for antimicrobial test. Wells containing serial dilution of DMSO and broth media only were also included as controls. After incubation at 37 °C for 24 and 48 h, the MIC was measured. Clear wells with the lowest concentration of extracts were taken as MIC values. 

**Phospholipid/polydiacetylene vesicle colorimetric assay**

To specifically detect membrane-active metabolites of the isolated fraction, a phospholipid/polydiacetylene vesicle was used as the membrane model^[^^21^^]^. To prepare the polymerized vesicles, dimyristoyl phosphatidylcholine and diacetylenic monomer 10,12-tricocosadiynoic acid were first separately dissolved in 1 mg/mL dichloromethane, and mixed at 2:3 molar ratio to a final concentration of 1 mM. The resulting vesicle solution had an intense blue color due to polymerization of the diacetylene^[^^22^^]^. The test solution was prepared by mixing 300 µL of the vesicles, 2 mM of Tris (pH 8.5), and the bioactive extracts to the final volume of 1 mL and finally incubated at 28 °C for one hour. Amphotericin B and tetracycline were used as positive and negative controls, respectively. Membrane specific activity was detected by the extent of blue-red color transition that was visible to the naked eyes.

**Circular dichroism** **spectroscopy**

The circular dichroism experiments were performed using a Jasco J-810 spectropolarimeter (Jasco Inc., USA). Different spectra between 190 to 260 nm were measured in a 1-mm long cell at room temperature. Data were recorded at 1 nm with a scan rate of 100 nm/min with a time constant of 4 s, and the average of three separate recordings with different concentrations of 1, 1:5, and 1:250 were analyzed^[^^23^^]^.

**Thin layer chromatography (TLC) analysis**

TLC was used to separate the compounds present in the crude ethyl acetate extract of the *Aspergillus* strain. The extract was loaded on thin-layer silica gel plates (Silica gel F254, Merck, Germany), and separated on TLC plates through different solvent systems. The crude extract was spotted, and the solvent front was allowed to be developed. The running lane was then dried to separate the bioactive compounds. The chromatogram was also visualized under a UV light system at 254 nm and 366 nm in the TLC chamber. The best mobile phase was selected according to spot resolution. The Rf value of each band was calculated as the distance traveled by the solute divided by the distance traveled by the solvent. Each fraction was further purified using preparative thin-layer chromatography (PTLC) and HPLC^[^^24^^]^.

**Purification by PTLC**

The preparation of slurry for coating the TLC plates was carried out through mixing silica gel 60 (GF254 Merck for PTLC) and water. This mixture was subsequently spread as thick slurry on a clean glass plate measuring 20 × 20 cm. The plate was dried and activated by heating in an oven at 110 °C for 30 min, then the sample was loaded on the plate. The choice of eluent was 1:1 n-hexane-ethyl acetate and was determined by preliminary analytical TLC. After elution in glass tanks, the silica gel regions containing the bands were scraped off the plate with a spatula. Each band was extracted with methanol, the silica residue was removed by centrifugation, and the supernatant was transferred to a vial. The individual metabolites were again spotted on a TLC plate to confirm the purity for each isolated metabolite.

**Compound characterization**

***HPLC analysis***

The HPLC system used was the Perkin Elmer model Flexar consisting of an injection valve with a 20-μL loop, and C18 column (10 mm × 150 mm) packed with silica gel. The column was equipped with a guard column C18 (4.6 mm × 150 mm), and the spectrophotometric detector was a diode-array detector (DAD), which plotted the detector outputs. The reagents for the mobile phase preparation were of the HPLC grade. For quantitative analyses, the mobile-phase consisted of acetonitrile and water in varying proportions. The gradient system was set as follows: first eight min with 20% water, next three min with 50% water, five min with 100% water, and seven last min with 20% water. The total run time was 23 min, and the flow rate was 1 mL/min. The DAD could detect all characteristic UV wavelengths, but the highest absorption peak was seen at 254 nm. The pure fraction was fractionated in the same way. 

**Table1:** Antimicrobial activity against *C. albicans*, *E. coli*, and *S. aureus*

**Strain Code/media**	***C. albicans *** **ATCC 10231**			***E. coli *** **ATCC 29922**			***S. aureus *** **ATCC 25923**
	**MIC (mg/mL)**			**MIC (mg/mL)**			**MIC (mg/mL)**
	**24 h**	**48 h**		**24 h**	**48 h**		**24 h**
2 d10	0.25	0.50		0.50	0.50		0.50
Ketoconazol	0.015	0.015		*-*	*-*		*-*
AmB	<0.007	0.007		*-*	*-*		*-*
Streptomycin	*-*	*-*		0.062	0.062		0.050
Kanamycin	*-*	*-*		*-*	*-*		0.025
DMSO v/v	5%	10%		>10%	>10%		5%

***Nuclear magnetic resonance spectroscopy*** ***(NMR) spectroscopy***

^1^H NMR (proton NMR) and H–H COSY spectra were recorded on Bruker 300 MHz spectrometer (Bruker Avance 300, Germany) in the MeOD-d4 solvent. All 1D and 2D spectra were obtained using the XWIN-NMR version 3.1. Chemical shifts were measured in parts per million (ppm) and coupling constants in Hertz (Hz)^[^^25^^]^.

***Liquid chromatography-mass spectrometry (*** ***LC-MS) analysis***

LC-MS was performed using Agilent Technologies 6410 Triple quad LC/MS, LC 1200 series, USA. Water and acetonitrile were used as mobile phases A and B, respectively. The gradient was set as first eight min with 80% A and 20% B, three min with 50% A and 50% B, five min with 100% B, and last seven min with 80% A and 20% B. The total run time was 23 min. The column used was C180, flow rate was 1 mL/min, DAD detector was set at 254 nm, and electrospray ionization was used in the positive mode^[^^26^^]^.

## RESULTS

According to our previous study on an *Aspergillus* strain isolated from the soil, we identified one strain producing membrane-active metabolites^[^^11^^]^. The ethyl acetate extract of the 2d10 strain showed activity against the indicator microorganisms (*E. coli*,* C. albicans, *and* S. aureus*). The evaluation of the antimicrobial activity of the isolate is summarized in Table 1. The results indicated that the selected strain produced metabolites that inhibited the growth of bacteria and yeast used in the assay.

**Morphological analysis**

**Fig. 1:**
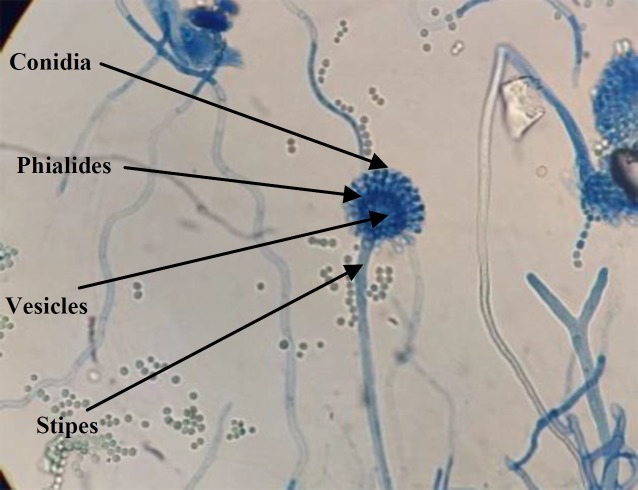
Microscopic feature of *A. flavus* on the Czapek Dox agar media. (magnification 40 ).

The microscopic characteristics of the conidiophore, stipe, and conidia of the *Aspergillus* isolate indicated similarities to *Aspergillus flavus* (Fig. 1).

**Characterization of** ***Aspergillus*** **strain**

***DNA sequencing***

**Fig. 2:**
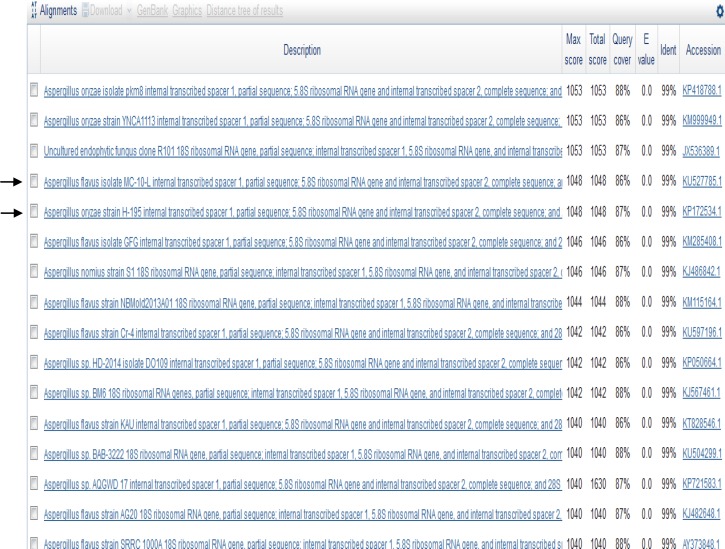
Comparative analysis of the ITS4 sequence in the GenBank database. The query *Aspergillus* strain had the same percentage of identity for both A. *flavus* and *A. oryzae*

The PCR product obtained from *Aspergillus* was identified to be about 598 bp long. The comparison of the ITS4 and ITS5 sequences and the 16S rRNA (598 bp) to the GenBank database revealed that the *Aspergillus* strain had sequences matching both *A. flavus* and *A. oryzae* (Fig. 2). Therefore, we sequenced the Bt2a and Bt2b gene fragment for a more specific comparative sequence analysis, which revealed that the *Aspergillus* strain had a 99% similarity to *A. flavus* (accession number kf434080.1).

**Colorimetric assay using artificial vesicles**

**Fig. 3:**
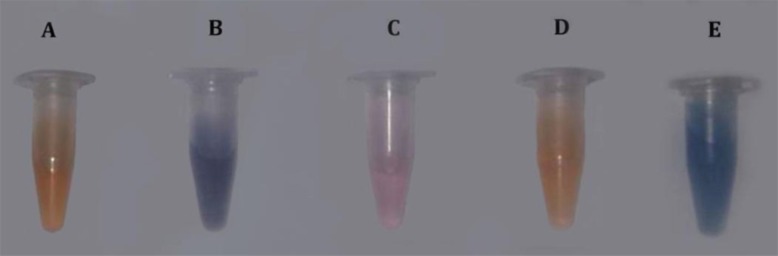
Artificial membrane acting assay for the selection of extracts and fractions that are membrane active. The results are indicated by a color change in the microvesicles made of lipid/polydiacetylene (PDA). Blue is inactive, and pink/red/orange colors show positive membrane activity. (A) 2d10, (B) tetracycline,(C) dimethyl sulfoxide, (D) amphotericin B, and (E) vesicle

Blue to red color transition was seen with the ethyl acetate fraction of *Aspergillus* strain 2d10. Amphotericin B was used as a positive control and tetracycline as a negative control. The color transition was visible with the naked eye and also confirmed by circular dichroism spectroscopy (Fig. 3).

**Circular dichroism and absorption measurements**

**Fig. 4:**
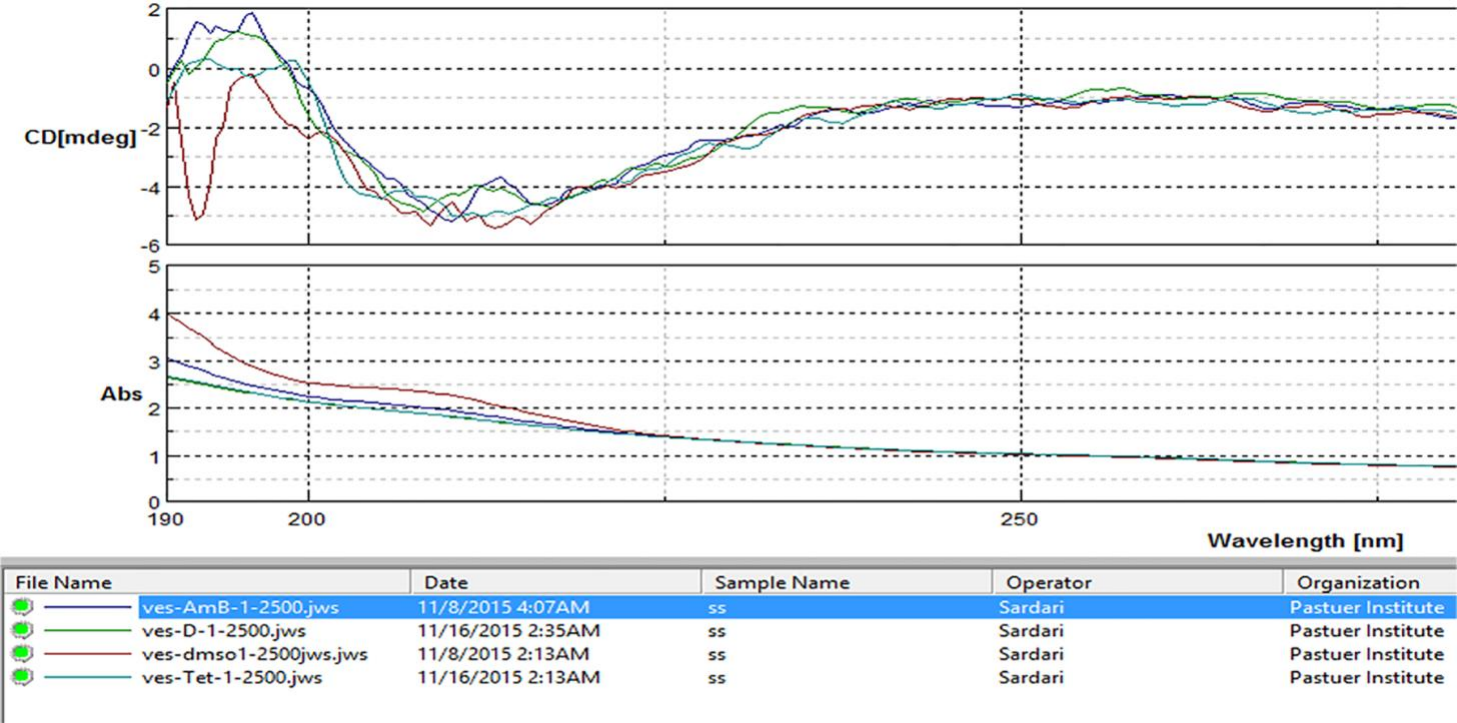
Circular dichroism and absorption measurements for membrane activity. The ethyl acetate fractions were named (D) 2d10. The concentration of solutions was diluted 2500 times to prevent detector saturation and noise

Color transition was seen with all dilutions (1, 1:5, 1:250, 1:2500) of the extract, and the circular dichroism absorbance values were not significantly different. In contrast, the absorbance of the control group (vesicle only) was significantly different from the other groups. These changes are consistent with the macroscopic color change as well as the UV absorbance (Fig. 4).

**Purification of metabolites by TLC and PTLC**

The best mobile phase for purifying the ethyl acetate fraction of *Aspergillus* 2d10 was a 50:50 mix of ethyl acetate and hexane. With this solvent system, TLC showed three metabolites with Rf values of 0.22, 0.35, and 0.42. The same mobile phase was used for PTLC to further separate the ethyl acetate fraction, which yielded purified secondary metabolites.

**Metabolite profile by HPLC analysis and LC-MS**

**Fig. 5:**
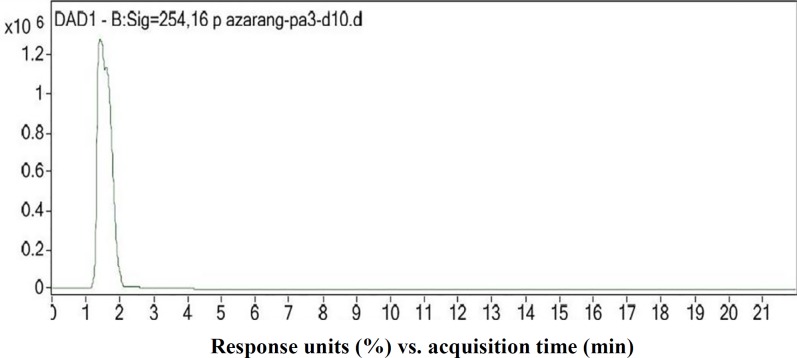
HPLC profile of the purified PA3-d10 compound

The HPLC profile of the ethyl acetate fraction was obtained at 254 nm, and the purity of the bioactive compound was analyzed. The chromatogram is shown in Figure 5. A sharp single peak was seen at the retention time of 1.537 min, which corresponded to the isolate named PA3-d10. The MS spectrum of the purified PA3-d10 compound at positive ion mode, and the electrospray ionization spectrum showed major peaks at 413 (m/z), indicating that the molecular mass of the purified compound corresponded to 413 (m/z).

**NMR analysis**

**Fig. 6:**
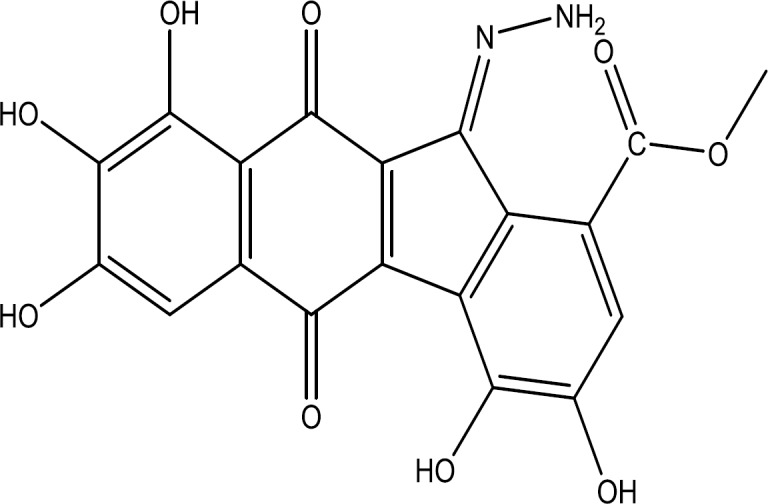
Suggested structure of the compound PA3-d10, Methyl (E)-11-hydrazineylidene-3,4,7,8,9-pentahydroxy-5,10-dioxo-10, 11-dihydro-5H-benzo[b]fluorene-1-carboxylate

^1^H-NMR of the purified compound was obtained at 300 MHz. The spectral chemical shifts in 2D COSY experiments were seen in MeOD-d4. Inter-molecular cross peaks were not observed. ^1^H NMR (300 MHz, MeOD-d4) result indicated the following peaks: δH (ppm) 3.30 (3H, s, O-CH_3_), 4.53 (2H, s, NH_2_), 6.49 (1H, s, H-Ar), and 7.93 (1H, s, H-Ar). These chemical signatures were obtained from the UV, LC-MS, ^1^H-NMR spectroscopy, and COSY indicated the structure of PA3-d10 (Fig. 6) along with the predicted peak positions obtained by ChemDraw Ultra 16.0, which were in agreement with the NMR results. The molecular formula of the purified bioactive compound was determined to be C_19_H_12_N_2_O_9_.

## DISCUSSIONS

Most secondary metabolites or antibiotics cannot be fully synthesized due to their complex structures and the high costs at an industrial scale. There is an urgent need for alternative biologically active secondary metabolites from various sources like plants, animals, or microorganisms^[^^27^^-^^29^^]^.

We identified a new strain of *Aspergillus* from Iranian soil and found that it produces novel membrane active secondary metabolites. The biological activity of the extract was shown on an artificial vesicle membrane system using circular dichroism, and alteration in membrane conformation was observed by colorimetric and spectroscopic changes. The membrane is a promising new target for antibiotic action. Various chromatographic techniques were then used to fractionate and purify the biologically active compounds from the fungal extract. PTLC and HPLC are the most widely used separation techniques used to characterize organic chemicals. A sensitive and rapid method is required to estimate the presence of secondary metabolites in the ethyl acetate extract of *Aspergillus*. To purify those metabolites, HPLC was applied using DAD detectors. We successfully combined PTLC and HPLC to isolate and characterize the biologically active secondary metabolites from the fungal sample, by recording chromatographic retention times and LC-MS spectra. The search of databases like ChEBI confirmed that PA3-d10 is a new natural product of the hydrazineylidene and benzo- naphthacenequinones families. In addition, many studies have reported the synthesis of these compounds as target structures and evaluated their biological activities^[^^30^^]^. Anti-microbial activities of these compounds have also investigated in other study^[^^32^^]^. Heinisch *et al.*^[^^31^^]^ have reported new benzo-naphthacene quinones derivatives that were screened against bacteria and showed appreciable antibacterial effects. These observations have been managing for the development of new hydrazones possessing various biological activities. Our study was able to identify novel antimicrobial compounds that act via disrupting membranes.

The present study was an attempt to identify membrane active fraction and to pick out strains that display antimicrobial activity against a variety of microbial pathogens. Membrane active fraction produced by *Aspergillus* strain in this research demonstrated antimicrobial activities against bacteria and yeast strains. Therefore, this compound can be used as an antimicrobial membrane active agent. In continuation of this research, the synthesis of derivatives and similar isolated compounds is underway.
